# Experimental Investigation on the Nonlinear Coupled Flutter Motion of a Typical Flat Closed-Box Bridge Deck

**DOI:** 10.3390/s20020568

**Published:** 2020-01-20

**Authors:** Guangzhong Gao, Ledong Zhu, Feng Wang, Hua Bai, Jianming Hao

**Affiliations:** 1 Highway College, Chang’an University, Xi’an 710064, China; 2 State Key Laboratory of Disaster Reduction in Civil Engineering, Tongji University, Shanghai 200092, China; 3 Key Laboratory of Structure and Wind Tunnel of Guangdong Higher Education Institutes, Shantou 515063, China; 4 Department of Bridge Engineering, Tongji University, Shanghai 200092, China; 5 Key Laboratory of Transport Industry of Bridge Wind Resistance Technology, Tongji University, Shanghai 200092, China

**Keywords:** nonlinear aeroelasticity, post flutter, limit cycle oscillation, closed box bridge section, wind tunnel

## Abstract

The nonlinear post-flutter instabilities were experimentally investigated through two-degree-of-freedom sectional model tests on a typical flat closed-box bridge deck (width-to-depth ratio 9.14). Laser displacement sensors and piezoelectric force balances were used in the synchronous measurement of dynamic displacement and aerodynamic force. Beyond linear flutter boundary, the sectional model exhibited heave-torsion coupled limit cycle oscillation (LCOs) with an unrestricted increase of stable amplitudes with reduced velocity. The post-critical LCOs vibrated in a complex mode with amplitude-dependent mode modulus and phase angle. Obvious heaving static deformation was found to be coupled with the large-amplitude post-critical LCOs, for which classical quasi-steady theory was not applicable. The aerodynamic torsional moment and lift during post-critical LCOs were measured through a novel wind-tunnel technique by 4 piezoelectric force balances. The measured force signals were found to contain significantly higher-order components. The energy evolution mechanism during post-critical LCOs was revealed via the hysteresis loops of the measured force signals.

## 1. Introduction

Flutter is the most dangerous aeroelastic instability for modern long-span bridges. Flutter instability is conventionally treated as a linear eigenvalue problem by classical linear flutter theory [1]. According to the linear theory, flutter instability occurs when the real part of a complex eigenvalue becomes negative and the vibration manifests as an exponential increase of vibration amplitude with time. Classical linear theory is based on a small-amplitude assumption and ignores any possible aerodynamic nonlinearity under large amplitude; therefore, the predicted flutter is an exponential-divergent type instability, which is also called ‘hard’ flutter, since the vibration amplitude suddenly increases to infinity beyond linear flutter boundary [2,3].

Experimental and numerical evidences suggest that the aerodynamic nonlinearity under large amplitude will introduce a secondary stabilizing effect and the flutter performance manifests as a soft-type nonlinear flutter instability [2,3,4,5,6,7,8,9,10,11,12,13,14]. When wind velocity exceeds beyond linear flutter boundary, there is a possible existence of nonlinear post-critical limit cycle oscillation (LCOs) due to the aerodynamic nonlinearity. Although the occurrence of flutter instability is strictly prohibited by the current wind-resistant standards, it is worthwhile to investigate the nonlinear post-flutter behaviors of common bridge decks when we try to reduce the potential of progressive collapse of long-span bridges under super-strong wind conditions, which correspond to extraordinarily high return periods in the lifetime. Moreover, modeling aerodynamic nonlinearity is beneficial to improve the accuracy of analytical precision of wind-induced vibration and as a result guarantees the structural safety and robustness of long-span bridges.

The aerodynamic nonlinearities in post-critical states have attracted wide attention in recent years [2,3,4,5,6,7,8,9,10,11,12,13,14]. The reported post-flutter phenomena vary with different deck shapes. Matsumoto et al. [3] tested the torsional flutter behaviors of H-shaped cylinders and found that the torsional flutter of relatively bluff H-shaped sections (width-to-depth ratio <3.4) exhibits non-divergent-type instability in a restricted velocity range, which seems to be influenced by vortex-induced vibration. When the width-to-depth ratio is further increased above 3.4, divergent-type instabilities were observed. Daito et al. [4] investigated the flutter instability of two-edge girders, where the geometrical shapes of edge girders were I-type, box-type and circular-type, respectively. The torsional flutter was found to be a non-divergent type with a gradual increase of torsional amplitude with velocity beyond flutter onset velocity for three different geometrical shapes of edge girders. Recently, the nonlinear post-flutter behaviors of two-edge girders were also extensively studied by Zheng et al. [5] and Tang et al. [6]. Zheng et al. [5] investigated the aerodynamic mitigation measures of the soft-type flutter of a Π-shaped bridge deck. They found that the post-flutter vibration amplitude was sensitive to the wind fairings and auxiliary facilities. Tang et al. [6] studied the post-critical response of a shallow Π shaped-section and found that the stable LCO amplitudes were sensitive to wind attack angle and structural damping. Gao et al. [7] studied the post-flutter behavior of a twin-side-girder bridge deck and found the sectional model exhibits significant post-critical LCOs with very slight heave-torsion coupling effects. A nonlinear 1-degree-of-freedom (DOF) empirical model was then proposed based on the measured self-excited torsional moment. 

The above-mentioned sections generally have a bluff aerodynamic configuration. Therefore, their nonlinear post-flutter behaviors mainly manifest as a torsional LCO with a relatively slight coupling of heaving DOF. However, for a quasi-streamlined section, the coupling of heaving DOF were found to be significant. Amandolese et al. [8] and Pigolotti et al. [9] investigated the post-critical behaviors of a thin plate and found significant heave-torsion coupled LCOs beyond linear flutter boundary. Amandolese et al. [8] also discussed the nonlinear hysteresis behavior around linear flutter boundary where the stable amplitudes depend on initial perturbations. Náprstek et al. [10] measured the nonlinear aeroelastic responses of several bluff bridge sections around linear flutter boundary by using a new mechanical device, and large-amplitude post-critical LCOs were observed in the torsional mode. Their mechanical device allows to mechanically decouple the heaving and torsional DOFs under excessively large amplitudes, and the structural damping can be mechanically adjusted. The nonlinear post-flutter behaviors of a closed-box section were found to exhibit a heave-torsion ‘soft’ flutter by Ying et al. [11] and Gao et al. [12]; the stable amplitudes of post-critical LCOs were found to be independent of initial perturbations, which is different from a thin plate. Wu et al. [13] tested the nonlinear flutter phenomenon of a truss bridge deck of Yang-Sigang Bridge. They found that a truss bridge deck could also exhibit a nonlinear soft flutter with a significant heave-torsion coupling effect. The coupling of heaving DOF provides negative damping, which will reduce the flutter onset wind speed and increase vibration amplitudes.

However, the experimental and numerical results are still very rare for common bridge decks, in particular, a flat-closed box bridge deck, which is a basic deck shape for many long-span bridges, such as Sutong Bridge, Taizhou Bridge, Lingdingyang Bridge, etc. As a typical engineering case, the design scheme of the Lingdingyang Bridge located in a typhoon-prone area adopts a flat closed-box bridge deck (width-to-depth ratio as 12.4) with a cantilever horizontal plate at the wind fairing which will act as a maintenance passage. The flutter instability was found to be very sensitive to wind attack angle. Its flutter boundary was governed by the nonlinear soft flutter starting from a relatively low wind speed 74 m/s (mean velocity) under attack angle 1°, which is well below the flutter checking velocity 83.5 m/s. Whilst, other attack angles between −3°–3° exhibited a divergent-type flutter instability and their flutter onset speeds were larger than the flutter checking velocity. Questions still remain as to whether the observed post-flutter LCOs are acceptable or not. Undoubtedly, a nonlinear self-excited force model is needed based on detailed experimental evidence to further address such questions. 

There are still many unsolved problems related with the nonlinear post-flutter behaviors of a flat-closed box section. Up to now, it is still unclear how the coupling of the heave-torsion DOFs evolves with wind velocity and whether the concept of linear mode applies in post-flutter state. In addition, the numerical study by Zhang et al. [14] suggests a possible coupling of static deformation during large-amplitude vibration. However, little attention has been paid to this coupling phenomenon.

In the present study, the nonlinear post-flutter behaviors of a typical flat-closed box bridge deck were extensively investigated through a series of wind-tunnel sectional model tests. Special attentions were paid to the nonlinear vibration mode and the coupling behavior of aerostatic deformation in post-critical states. A novel wind-tunnel technique was employed to measure the nonlinear self-excited force in sync with the recording of dynamic displacement. Based on the measured force signals, the underlying aerodynamic nonlinearities and energy evolving mechanism during post-flutter states were discussed.

## 2. Wind Tunnel Tests

### 2.1. Experimental Setup

**Figure 1:**
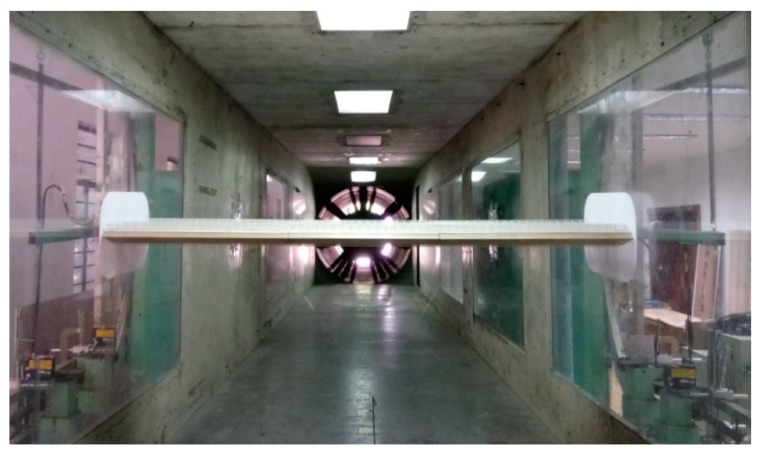
View of the spring-suspended sectional model in TJ-1 wind tunnel.

**Figure 2:**
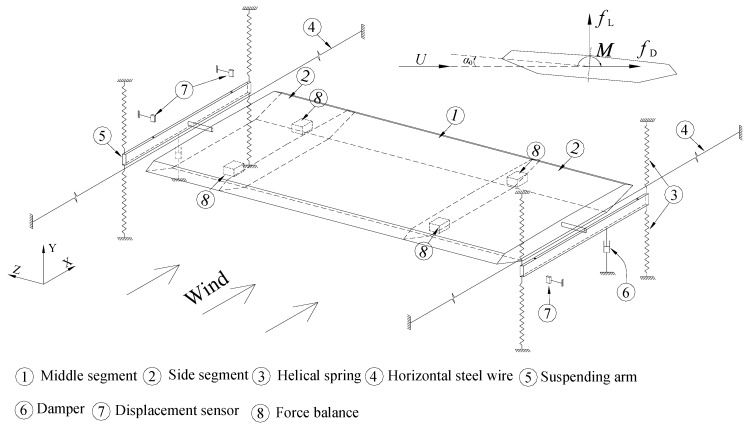
Schematic view and notations of the aeroelastic setup (end plates in Figure 1 not shown).

Sectional model tests were performed in TJ-1 Wind Tunnel, which is a sucking-type open-circuit wind tunnel located in Tongji University. The test section is 1.8 m (width) × 1.8 m (height). As shown in Figure 1, the sectional model was placed horizontally in the test section with its longitudinal axis perpendicular to the oncoming flow. Two end plates (600 mm × 300 mm) were attached to both ends to suppress axial flow through the holes of wind tunnel walls. The model was supported by 8 helical springs through 2 suspending arms. A pair of long-tensioned steel wires was used to constrain the horizontal motion of the sectional model as illustrated in Figure 2. Steel-wire-rope dampers were installed together with helical springs to adjust structural damping; one can refer to Reference [15] for more details of the damper. All helical springs, suspending arms, dampers and displacement sensors were placed outside of wind tunnel walls to avoid any interference on the flow field. 

The heaving motion *h*(t) and torsional motion *α*(t) were measured by 4 laser displacement sensors (Panasonic HL-G112-S-J shown in Figure 18a) with a linear measurement range of ±60 mm. A total of 4 piezoelectric force balances were installed inside the model to measure the transient aerodynamic force as shown in Figure 2 and Figure 18b,c, which will be introduced in the next section. The mean wind velocity was measured by a pitot-static tube placed in the upstream. The dynamic displacement sensors and force balances were connected to a 24 bits resolution acquisition system. The sampling frequency was chosen as 200 Hz.

**Figure 3:**
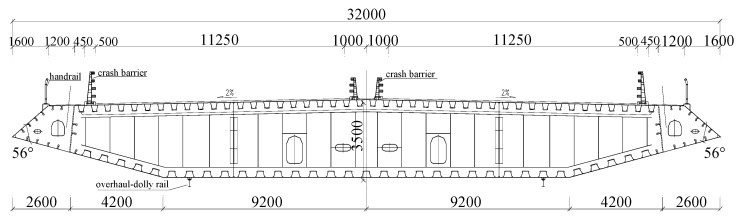
The flat closed-box bridge deck (unit: mm).

All tests were carried out in a nominal smooth flow field with a background turbulence intensity *I*_u_ < 1%. The tested mean velocity *U* was in the range of 1 m/s–16.5 m/s, and the corresponding Reynolds number *Re* = *ρUB*/*μ* was 3.29 × 10^4^–5.42 × 10^5^ being *B* the width of the sectional model. The configuration of the cross section is presented in Figure 3, which is the original design scheme of Xiangshan Harbor Bridge in China. The model was manufactured elaborately in the light of the geometric similarity principle and the length scale was chosen as 1:65; as a result, the width *B* and depth *D* of the sectional model were 0.4923 m and 0.0538 m, respectively. The model length *L* is 1.760 m. The side ratio *B*/*D* and aspect ratio *L*/*B* of the sectional model were 9.14 and 3.57, respectively. The blockage ratio *D*_0_*/H*_wt_ was about 3% and 3.8%, respectively, for static attack angle 0° and 3°, being *D*_0_ the effective windward height of the model and *H*_wt_ the height of the wind-tunnel test section.

All test configurations are listed in Table 1, among which particular attention was paid to study the influence of attack angle and structural damping. The static attack angle *α*_0_ was adjusted by an angle control device connected to the 8 helical springs. The structural damping and frequency were measured by the logarithmic decrement method from the free decay responses in still air.

### 2.2. Linear Aeroelastic Property

The oscillating sectional model immersed in a smooth flow field is subjected to self-excited loads. The governing equations can be established around the static equilibrium positions as

(1a) $$m\left(\ddot{h}+2\xi_{h0}\dot{h}+\omega_{h0}^{2}h\right)=L_{\mathrm{se}}\left(h,\;\dot{h},\;\ddot{h},\;\alpha ,\;\dot{\alpha },\ddot{\alpha }\right)$$

(1b) $$J_{m}\left(\ddot{\alpha }+2\xi_{\alpha 0}\dot{\alpha }+\omega_{t0}^{2}\alpha \right)=M_{\mathrm{se}}\left(h,\;\dot{h},\;\ddot{h},\;\alpha ,\;\dot{\alpha },\ddot{\alpha }\right)$$

where $L_{\mathrm{se}}$ and $M_{\mathrm{se}}$ are self-excited lift and torsional moment per unit length, respectively. Both $L_{\mathrm{se}}$ and $M_{\mathrm{se}}$ are dependent on the heave-torsion vibration responses.

Self-excited loads have aerodynamic stiffness and damping effects, which lead to reduced-velocity dependence of the vibration frequency and damping ratio. To quantify the aerodynamic stiffness and damping effect, free decay tests were performed for various velocities by applying a coupled heave-torsion perturbation to the sectional model. The modal frequencies and damping ratios were then identified from the free decay responses by the logarithmic decrement method. For unstable conditions, the model was manually stabilized at first around its zero position and then let free to vibrate. The build-up vibration response in small amplitude regime was employed in identifying modal frequencies and damping ratios.

**Figure 4:**
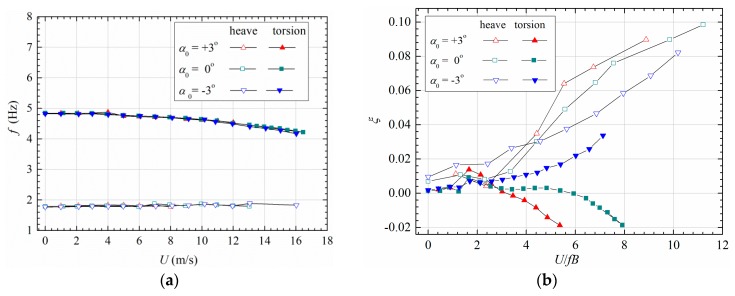
Evolution of linear modal parameters with reduced velocity: (**a**) frequency, (**b**) damping (Case A1, B1 and C1).

The identified linear modal frequencies and damping ratios are plotted in Figure 4. One can find that the model vibrated in heaving and torsional modes. In Figure 4a, the heaving frequency slightly increases with velocity, and the torsional frequency smoothly decreases. Figure 4b shows that all heaving modes for different attack angles are stable with monotonously increasing damping ratio with reduced velocity. The torsional mode under attack angle −3° is also stable, whereas those of attack angle 0° and 3° become unstable in large reduced velocity. Because the torsional damping ratios decrease and cross zero axis at around a critical velocity $U_{\mathrm{cr}}^{*}$ = 3.01 and 5.38, respectively, for attack angle 0° and 3°. The critical velocity $U_{\mathrm{cr}}^{*}$ represents a boundary where the torsional mode changes its stability; therefore, it is also called as flutter onset wind speed or linear flutter boundary. Moreover, note that the torsional damping of attack angle −3° would also become negative when wind velocity is sufficiently high, whereas the lower flutter boundary is the main concern in engineering application.

The aeroelastic loads are conventionally approached by a linearized theory proposed by Scanlan and Tomko [1], which simplifies the complex wind-structure interaction by the following linear self-excited force model

(2a) $$L_{\mathrm{se}}=\frac{1}{2}\rho U^{2}\left(2B\right)\left[KH_{1}^{*}\left(K\right)\frac{\dot{h}}{U}+KH_{2}^{*}\left(K\right)\frac{B\dot{\alpha }}{U}+K^{2}H_{3}^{*}\left(K\right)\alpha +K^{2}H_{4}^{*}\left(K\right)\frac{h}{B}\right]$$

(2b) $$M_{\mathrm{se}}=\frac{1}{2}\rho U^{2}\left(2B^{2}\right)\left[KA_{1}^{*}\left(K\right)\frac{\dot{h}}{U}+KA_{2}^{*}\left(K\right)\frac{B\dot{\alpha }}{U}+K^{2}A_{3}^{*}\left(K\right)\alpha +K^{2}A_{4}^{*}\left(K\right)\frac{h}{B}\right]$$

where ρ is air density. K=ωB/U is reduced frequency. $H_{1}^{*}$,$H_{2}^{*}$,$H_{3}^{*}$,$H_{4}^{*}$,$A_{1}^{*}$,$A_{2}^{*}$,$A_{3}^{*}$ and $A_{4}^{*}$ are flutter derivatives, which are dependent on reduced frequency K to consider unsteady effect.

**Figure 5:**
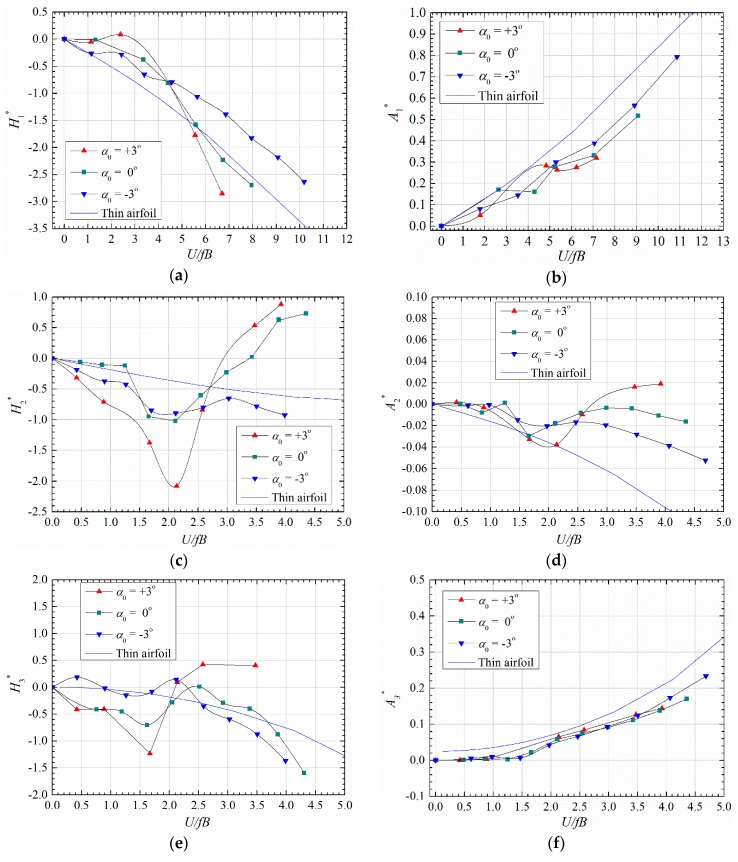
Identified linear flutter derivatives for different attack angles (Case A1, B1 and C1). (**a**) $H_{1}^{*}$; (**b**) $A_{2}^{*}$; (**c**) $H_{2}^{*}$; (**d**) $A_{2}^{*}$; (**e**) $H_{3}^{*}$; (**f**) $A_{3}^{*}$.

The flutter derivatives of the sectional model were identified by the revised MLS method proposed by Ding et al. [16]. The identified results are plotted in Figure 5, and the theoretical values of a thin airfoil calculated by Theodorsen’s theory are also presented for comparison [1,17]. 

One can notice from Figure 5 that the flutter derivatives vary significantly with initial attack angles indicating that the aeroelastic behavior is sensitive to wind attack angle. The slope of damping coefficient $A_{2}^{*}$ becomes positive at large reduced velocity for attack angle 0° and 3°, which destabilizes the torsional mode and leads to the negative torsional damping in Figure 4b together with the coupling term $A_{1}^{*}H_{3}^{*}$.

### 2.3. Bifurcation beyond Linear Flutter Boundary

**Figure 6:**
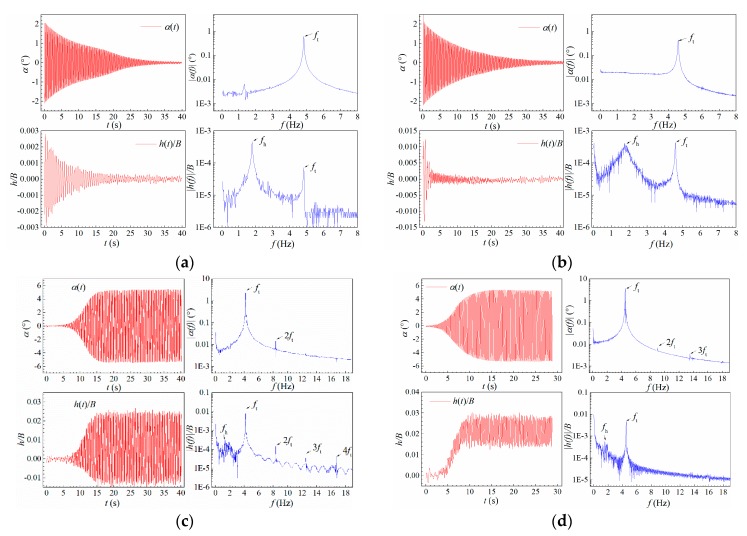
Typical vibration responses under different reduced wind speed. (**a**) Case B1, U* = 0.855; (**b**) Case B1, U* = 4.835; (**c**) Case B1, U* = 7.785; (**d**) Case A1, U* = 5.365.

Figure 6 shows the typical vibration phenomena of the sectional model. Blow linear flutter boundary $U_{\mathrm{cr}}^{*}$, the model vibration was stable around its static equilibrium position. Any initial perturbation will decay to a random small-amplitude vibration as shown in Figure 6a,b. The heaving components decay more rapidly for higher reduced velocity since the heaving damping increases as in Figure 4b. There always exist two peaks in the amplitude spectrum of heaving vibration, which correspond to the heaving mode and torsional mode.

When reduced velocity increased beyond linear flutter boundary, the model lost its stability around static equilibrium position. The amplitudes of heaving and torsional vibration both increased with time exhibiting an obvious heave-torsion coupling effect. The aerodynamic nonlinearity has a stabilizing effect, which reduces the increase rate of amplitude. The post-critical instability thus manifested as heave-torsion LCOs as in Figure 6c,d. One can find from the amplitude spectra in Figure 6c,d that only the torsional mode increased during post-critical LCOs whilst the component of heaving mode did not change when compared with Figure 6a,b. Therefore, the post-critical LCO occurred in the torsional mode. Moreover, note that both the heaving and torsional displacement responses are not strictly harmonic, since their amplitude spectra contain slightly higher-order harmonic components due to aerodynamic nonlinearity.

Another noticeable phenomenon is that the post-critical LCOs were coupled with a significant drift of static equilibrium position. As can be seen in Figure 6c,d, the heaving static equilibrium position gradually moves upward along with the increase of vibration amplitude. There is no obvious change in the torsional static equilibrium position. Positive attack angle 3° corresponded to more significant drift of heaving static equilibrium position than attack angle 0°. This phenomenon was also reported by Zhang et al. [14] in a numerical study on a flat box bridge deck (side ratio *B*/*D* = 12.3) but not mentioned by Amandolese et al. [8] in studying the post-critical behavior of a thin plate. The heaving static deformation is probably because of the aerodynamic asymmetry induced by large-amplitude oscillations of instantaneous attack angle. This phenomenon will be discussed in Section 2.6.

**Figure 7:**
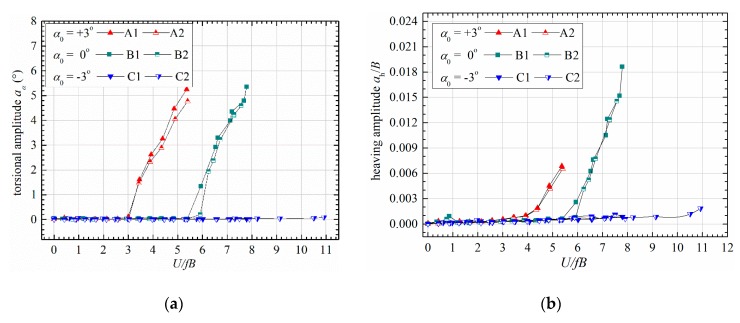
Evolution of stable amplitudes with reduced wind speed under different attack angles. (**a**) Torsional amplitude; (**b**) heaving amplitude.

Figure 7 shows the stable amplitudes of post-critical LCOs. One can find that the stable amplitudes of torsional and heaving LCOs both increased unrestrictedly with reduced velocity. Note that the recorded maximum amplitude is about 5.4°, which is limited by the linear range of displacement sensors. Increasing structural damping will reduce the stable amplitudes. Both the torsional and heaving amplitudes increase smoothly from zero positions with no obvious ‘sudden jump’ as reported in the tests of thin plates by Amandolese et al. [8] and Pigolotti et al. [9]. Therefore, the observed post-critical instability is a kind of nonlinear ‘soft flutter’. The stable amplitudes were also found to be independent of initial excitations, such as increasing or decreasing wind speed, perturbation amplitudes, etc.

**Figure 8:**
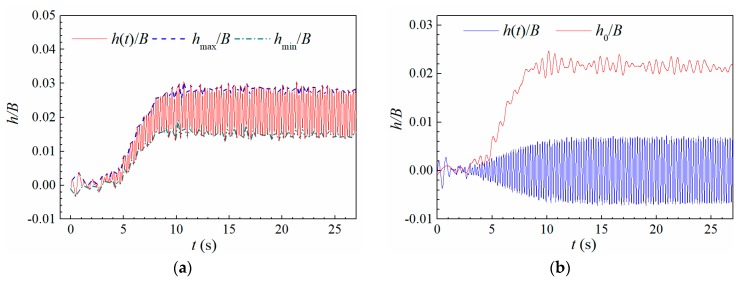
Separation of post-critical LCO and static deformation (Case A1, U* = 5.365) (**a**) Recorded heaving displacement; (**b**) pure heaving vibration and static displacement.

To facilitate further modeling of post-critical LCOs, the coupling of post-critical LCO and static deformation can be separated. The static deformation can be represented by the average value of upper and lower envelopes. The decoupling process is expressed as

(3a) $$h_{\mathrm{se}}\left(t\right)=h\left(t\right)-h_{0}\left(t\right)$$

(3b) $$h_{0}=\frac{h_{\max}+h_{\min}}{2}$$

where h(t) is recorded heaving displacement. $h_{\mathrm{se}}\left(t\right)$ is the pure post-critical LCO, and $h_{0}$ represents the heaving static deformation. $h_{\max}$ and $h_{\min}$ are the upper and lower envelopes, respectively. Figure 8 demonstrates the separated heaving LCO in Figure 6d.

As discussed earlier, the observed post-critical LCOs were featured by obvious heave-torsion coupling effect. The degree of heave-torsion coupling can be quantitatively represented by a coupling ratio [2]

(4) $$\gamma =\frac{h_{\mathrm{rms}}}{\alpha_{\mathrm{rms}}b}$$

where $h_{\mathrm{rms}}$ and $\alpha_{\mathrm{rms}}$ are respectively the root-mean-square values of heaving and torsional displacement in a steady-amplitude stage. b=B/2 is the half width of a bridge deck.

**Figure 9:**
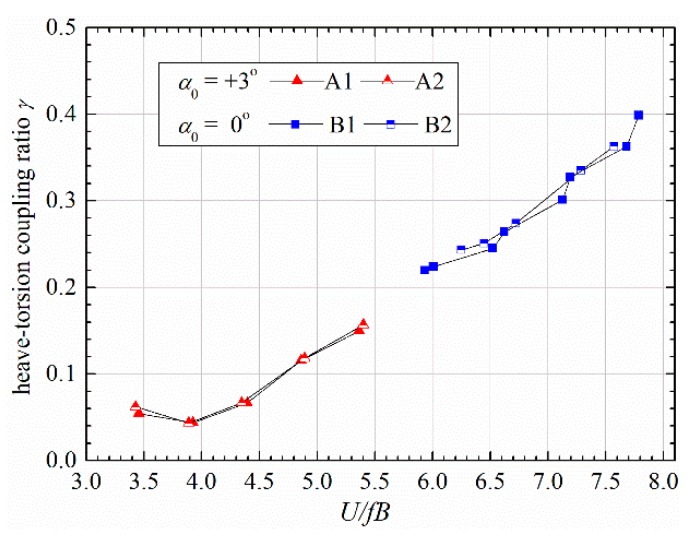
Heave-torsion coupling ratio versus reduced wind speed in post-critical state.

Figure 9 presents the coupling ratios of attack angle 0° and 3°. One can find that the heave-torsion coupling ratio increases approximately in a linear manner with reduced velocity, which is consistent with the test results on a larger side-ratio *B*/*D* = 10.7 [12]. The influence of structural damping on heave-torsion coupling is very slight and can be neglected. Static attack angle does not change the evolving trend of coupling ratio with reduced velocity; therefore, the relatively weak coupling in Figure 6 and Figure 7 for attack angle 3°, when compared with attack angle 0°, is mainly due to that its post-critical response lies in a relatively low range of reduced velocity. 

### 2.4. Vibration Mode during Post-Critical LCO

To investigate the vibration pattern during post-flutter instability, the applicability of linear mode is checked in the following. Firstly, the quasi-harmonic torsional and heaving displacement in the torsional mode during a post-critical LCO can be expressed as

(5a) $$\alpha \left(t\right)=a_{\alpha }\cos\left(\omega_{t}t+\beta_{\alpha }\right)$$

(5b) $$h\left(t\right)=a_{h}\cos\left(\omega_{t}t+\beta_{h}\right)$$

where $a_{\alpha }$ and $a_{h}$ are respectively the instantaneous torsional and heaving amplitude. $\beta_{\alpha }$ and $\beta_{h}$ are respectively the torsional and heaving phase. $\omega_{t}$ is the circular frequency of torsional mode.

According to classical linear flutter theory [1,16], the torsional and heaving vibration can be expressed by a linear torsional mode as

(6) $$\left[\begin{array}{c} h\left(t\right)\\ \alpha \left(t\right) \end{array}\right]=a_{0}\varphi_{t}e^{\left(-\xi_{t}\omega_{t}+i\omega_{t}\right)t}+a_{0}^{*}\varphi_{t}^{*}e^{\left(-\xi_{t}\omega_{t}-i\omega_{t}\right)t}$$

where the values with superscript * represent the corresponding complex conjugate. $a_{0}$ is a constant value related with the initial condition. $\xi_{t}$ is the torsional damping ratio. $\varphi_{t}$ is the vector of torsional mode

(7) $$\varphi_{t}\left(t\right)=\left[\begin{array}{c} U_{h2}+iV_{h2}\\ U_{\alpha 2}+iV_{\alpha 2} \end{array}\right]\;\underset{\to }{\mathrm{normalization}}\left[\begin{array}{c} U_{h2}^{\#}+iV_{h2}^{\#}\\ 1 \end{array}\right]$$

where i is the imaginary unit. $U_{h2}$, $V_{h2}$, $U_{\alpha 2}$ and $V_{\alpha 2}$ are parameters of the complex mode vector. $U_{h2}^{\#}$ and $V_{h2}^{\#}$ are parameters of normalized mode vector.

Substitute Equation (7) into Equation (6), yields

(8) $$\left[\begin{array}{c} h\left(t\right)\\ \alpha \left(t\right) \end{array}\right]=\frac{a_{\alpha 0}e^{i\beta_{\alpha }}}{2}\left[\begin{array}{c} U_{h2}^{\#}+iV_{h2}^{\#}\\ 1 \end{array}\right]e^{\left(-\xi_{t}\omega_{t}+i\omega_{t}\right)t}+\frac{a_{\alpha 0}e^{-i\beta_{\alpha }}}{2}\left[\begin{array}{c} U_{h2}^{\#}+iV_{h2}^{\#}\\ 1 \end{array}\right]e^{\left(-\xi_{t}\omega_{t}-i\omega_{t}\right)t}$$

where $a_{\alpha 0}$ is the initial torsional amplitude.

From Equation (8), we have

(9a) $$\alpha \left(t\right)=2\mathrm{Real}\left[\frac{a_{\alpha 0}e^{i\beta_{\alpha }}}{2}e^{\left(-\xi_{t}\omega_{t}+i\omega_{t}\right)t}\right]=a_{\alpha 0}e^{-\xi_{t}\omega_{t}t}\cos\left(\omega_{t}t+\beta_{\alpha }\right)$$

(9b) $$h\left(t\right)=2\mathrm{Real}\left[\frac{a_{\alpha 0}e^{i\beta_{\alpha }}}{2}\left(U_{h2}^{\#}+iV_{h2}^{\#}\right)e^{\left(-\xi_{t}\omega_{t}+i\omega_{t}\right)t}\right]=a_{\alpha 0}e^{-\xi_{t}\omega_{t}t}\sqrt{U_{h2}^{\#2}+V_{h2}^{\#2}}\cos\left(\omega_{t}t+\beta_{\alpha }+\Delta \beta \right)$$

(9c) $$\Delta \beta =\arctan\frac{V_{h2}^{\#}}{U_{h2}^{\#}}$$

where the function Real() means getting the real part of a complex value. Δβ is the phase difference between the heaving and torsional displacement. 

Compare Equation (9) with Equation (5); we obtain

(10a) $$\Delta \beta =\beta_{h}-\beta_{\alpha }$$

(10b) $$\frac{a_{h}}{a_{\alpha }}=\sqrt{U_{h2}^{\#2}+V_{h2}^{\#2}}$$

Equations (9c)–(10b) indicates that the torsional mode modulus $\sqrt{U_{h2}^{\#2}+V_{h2}^{\#2}}$ is identical to the heave-torsion amplitude ratio $a_{h}/a_{\alpha }$, and the mode phase angle equals the phase difference Δβ between heaving and torsional DOFs. Therefore, we can check the evolution of heave-torsion amplitude ratio $a_{h}/a_{\alpha }$ and phase difference Δβ during post-critical LCOs to check the applicability of classical linear mode in describing large-amplitude post-flutter instability.

**Figure 10:**
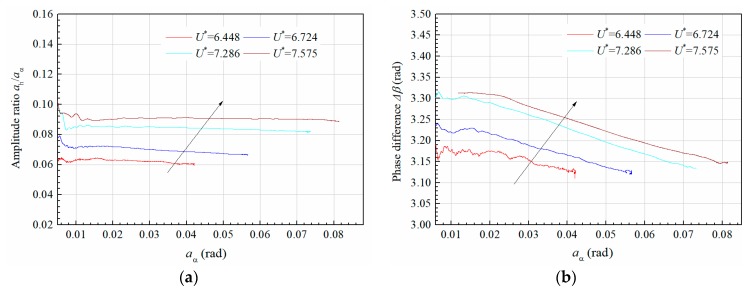
Influence of vibration amplitude on torsional mode (Case B2). (**a**) Mode modulus; (**b**) phase difference.

Figure 10 shows the evolution of heave-torsion amplitude ratio $a_{h}/a_{\alpha }$ and phase difference Δβ with torsional amplitude $a_{\alpha }$ during the whole post-critical LCOs under several reduced velocities. One can find that both the amplitude ratio $a_{h}/a_{\alpha }$ and phase difference Δβ increase with reduced wind velocity and slowly decrease with torsional amplitude. Therefore, the parameters of torsional mode ($U_{h2}^{\#}$ and $V_{h2}^{\#}$) are also be amplitude-dependent. A larger side ratio corresponds to a more significant amplitude-dependent effect of torsional mode, when compared with the previous study on a larger side-ratio *B*/*D* = 10.7 [12]. Also note that the calculated amplitude ratio and phase difference contain some high-frequency fluctuations; Figure 10 only plots the slow-varying trend by filter out the high-frequency components.

**Figure 11:**
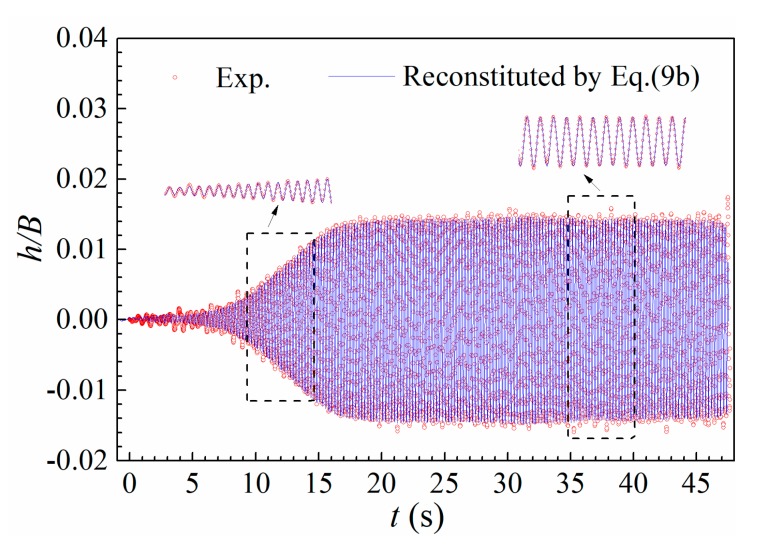
Comparison of a heaving LCO reconstituted from amplitude-dependent mode and experimental result (Case B2, U* = 7.575).

To verify the applicability of amplitude-dependent torsional mode in describing post-critical LCOs, the identified heave-torsion amplitude ratio $a_{h}/a_{\alpha }$ and phase difference Δβ plotted in Figure 10 was employed to calculated the amplitude-dependent mode parameters $U_{h2}^{\#}$,$V_{h2}^{\#}$ according to Equations (9c) and (10b). The obtained $U_{h2}^{\#}$ and $V_{h2}^{\#}$ were substituted into Equation (9b) to reconstitute heaving displacement h(t) from the recorded α(t). The comparison of the reconstituted heaving displacement and experimental result is shown in Figure 11. One can find that the reconstituted response is in good agreement with experimental result.

### 2.5. Amplitude-Dependent Damping and Frequency

**Figure 12:**
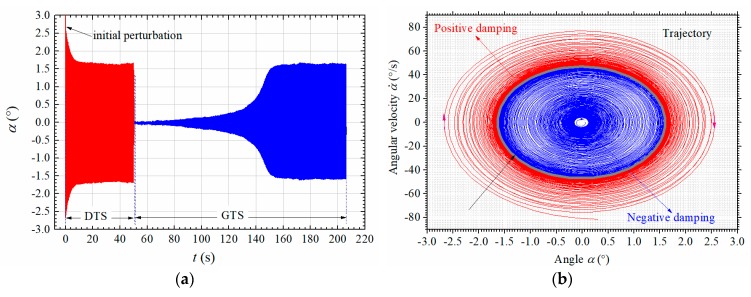
Typical post-critical LCO under different initial perturbations (Case A1, U* = 3.456). (**a**) Time history; (**b**) phase diagram.

Typical post-critical LCO under different initial perturbations is presented in Figure 12a. One can find that the vibration will decay to a stable amplitude (DTS) from a large initial perturbation and will grow to a stable amplitude (GTS) from the zero position. Figure 12b shows the corresponding phase diagram. The decay and building-up process both converge to a closed trajectory, which corresponds to the stable LCO amplitude. The closed trajectory is very close to an ellipse indicating weak nonlinearity.

The previous section has discussed the amplitude-dependence of torsional mode. Similarly, the damping ratio and frequency are also amplitude-dependent due to the existence of aerodynamic nonlinearity. The amplitude-dependence of torsional damping can be clearly inferred from Figure 12, because the amplitude change rate evolves for different vibration amplitude.

To identify the amplitude-dependent aerodynamic damping, the total damping ratio is expressed as

(11) $$\xi_{t}=\xi_{s}+\xi_{\mathrm{se}}$$

where $\xi_{t}$ is the total damping ratio of the torsional mode for any specific wind velocity. $\xi_{s}$ is structural damping ratio, which can be identified from the free-decay torsional response in still air. $\xi_{\mathrm{se}}$ is the aerodynamic damping ratio induced by self-excited force.

The total damping ratio can be identified from the time-varying torsional envelope by Equation (9a), which is

(12) $$a_{\alpha }\left(t\right)=a_{\alpha 0}e^{-\xi_{t}\omega_{t}t}$$

Calculating the logarithm on the both sides of Equation (12), yields

(13) $$\ln a_{\alpha }=\ln a_{\alpha 0}-\xi_{t}\omega_{t}t$$

Differentiating Equation (13) with respect to time, we have the amplitude-dependent damping ratio as

(14) $$\xi_{t}=\frac{d\left(-\ln a_{\alpha }+\ln a_{\alpha 0}\right)}{\omega_{t}dt}=-\frac{da_{\alpha }}{\omega_{t}a_{\alpha }dt}$$

The amplitude-dependent frequency can be identified from the zero-crossing time points as follows

(15) $$f_{t,i}=\frac{1}{2\left(t_{i}-t_{i-1}\right)},\;\;\alpha \left(t_{i}\right)=0$$

where $t_{i}$ is the time point where the torsional displacement α(t) crosses zero axis.

**Figure 13:**
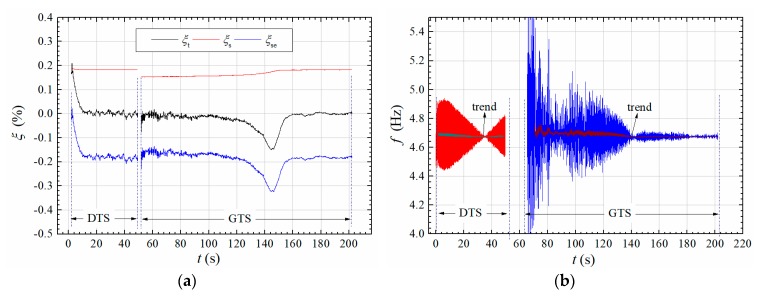
Evolution of amplitude-dependent (**a**) damping ratio; (**b**) vibration frequency during a post-critical LCO in Figure 12a (Case A1, U* = 3.456).

The amplitude-dependent aerodynamic damping ratio $\xi_{\mathrm{se}}$ can then be obtained by subtracting the structural damping $\xi_{s}$ from the total damping ratio calculated by Equation (14). Figure 13 shows the calculated time-varying damping ratios and frequency during the DTS and GTS processes in Figure 12. One can find from Figure 13a that the aerodynamic damping $\xi_{\mathrm{se}}$ is positive and rapidly decays to a negative value to balance the positive structural damping $\xi_{s}$ during the DTS process; during the GTS process, the aerodynamic damping $\xi_{\mathrm{se}}$ is negative in the initial stage and gradually decrease during the amplitude-growing stage when *t* < 146 s, but with the increase of amplitude, the aerodynamic damping $\xi_{\mathrm{se}}$ increases resulting in lower increasing rate of vibration amplitude in Figure 12a. Finally, $\xi_{\mathrm{se}}$ reaches a stable negative value balancing the structural damping $\xi_{s}$. Therefore, the post-critical LCOs are closely related with the amplitude-dependent effect of aerodynamic damping $\xi_{\mathrm{se}}$.

Figure 13b shows the identified time-varying vibration frequency during post-critical LCOs. Significant fluctuation can be observed in the identified result, which may be possibly due to the fact that the torsional vibration is not strictly symmetric about zero axis during the amplitude slow-varying process. We can further get the long-term trend of frequency series. One can then find that the evolution of the long-term trend is very slight along with the change of vibration amplitude in Figure 12a.

### 2.6. Coupling of Aerostatic Deformation and Large-Amplitude Vibration

**Figure 14:**
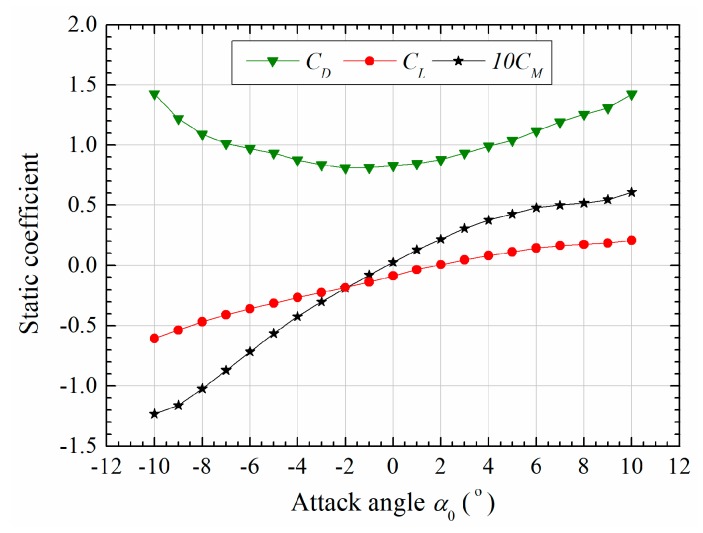
Steady force coefficients versus attack angles (*Re* = *ρUB*/*μ* = 4.656 × 10^5^) [18].

To investigate the coupling phenomenon of post-critical LCO and aerostatic deformation in Figure 6c,d, the aerostatic coefficients of the sectional model were firstly measured. The measurement of aerostatic coefficients was similar to the above-mentioned aeroelastic setup except that the elastically supported system was replaced by a rigidly fixture connected to the wind tunnel walls, and five-component strain-gauge balance was employed. The measured results are shown in Figure 14, where the coefficients are defined as

(16a) $$C_{D}=\frac{F_{D}}{1/2\rho U^{2}D}$$

(16b) $$C_{L}=\frac{F_{L}}{1/2\rho U^{2}B}$$

(16c) $$C_{M}=\frac{M}{1/2\rho U^{2}B^{2}}$$

where $F_{D}$, $F_{L}$, M represent respectively the aerodynamic drag force, lift force and torsional moment per unit length. $C_{D}$, $C_{L}$ and $C_{M}$ are aerostatic drag coefficient, lift coefficient and moment coefficient, respectively.

Aerostatic force will lead to static deformation in flowing air conditions. As a result, the torsional and heaving equilibrium positions will vary with wind speed. The resultant static attack angle and heaving deformation can be expressed as

(17a) $$\alpha_{0}^{*}=\alpha_{0}+\Delta \alpha_{0,S}$$

(17b) $$\Delta h_{0}=\Delta h_{0,S}+\Delta h_{0,L}$$

where $\Delta \alpha_{0,S}$ is the drift of static attack angle relative to still air position $\alpha_{0}$ because of aerostatic moment. $\alpha_{0}^{*}$ is the resultant static attack angle under flowing air conditions. $\Delta h_{0}$ is the total drift of the heaving zero position. $\Delta h_{0,S}$ is the drift of the heaving zero position relative to still air in small amplitude stage. $\Delta h_{0,L}$ is the additional drift of the heaving zero position as shown in Figure 8 along with the increase of vibration amplitude. Moreover, note that the additional drift of the torsional attack angle under large amplitude is negligible as indicated from Figure 6c,d.

According to Equation (16), the static deformation $\Delta \alpha_{0,S}$ and $\Delta h_{0,S}$ can be calculated as

(18a) $$\frac{1}{2}\rho U^{2}B^{2}LC_{M}\left(\alpha_{0}+\Delta \alpha_{0,S}\right)=k_{\alpha }\Delta \alpha_{0,S}$$

(18b) $$\frac{1}{2}\rho U^{2}BLC_{L}\left(\alpha_{0}+\Delta \alpha_{0,S}\right)=k_{h}\Delta h_{0,S}$$

where $k_{h}$ and $k_{\alpha }$ are respectively the elastic heaving and torsional stiffness of the spring-suspended system, which are expressed as

(19a) $$k_{h}=m\omega_{h}^{2}L$$

(19b) $$k_{\alpha }=\frac{k_{h}e^{2}}{4}$$

where e = 0.824 m is the distance between the fixed points of helical springs on each suspending arm in Figure 2.

**Figure 15:**
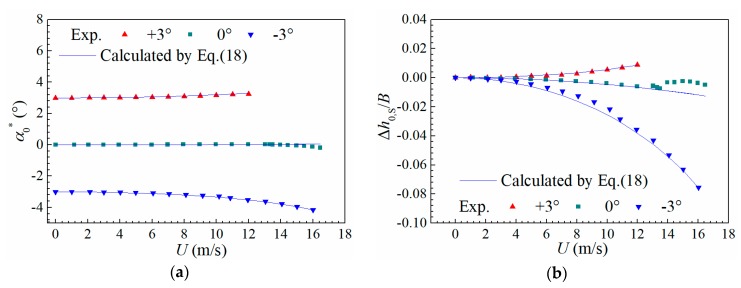
Static (**a**) torsional deformation and (**b**) heaving deformation of the sectional model versus wind speed.

Figure 15 shows the calculated static deformation $\Delta \alpha_{0,S}$ and $\Delta h_{0,S}$ along with the tested wind speed. One can find that the calculated results agree well with experiments, which indicates that the static drift of torsional and heaving equilibrium positions under small amplitude can be predicted by aerostatic coefficients with a satisfactory accuracy.

For the additional drift $\Delta h_{0,L}$ under large vibration amplitude, quasi-steady theory is firstly employed according to the Equation (18), which is

(20) $$k_{h}\left(\Delta h_{0,S}+\Delta h_{0,L}\right)=\frac{1}{2}\rho U^{2}BLC_{L}\left(\alpha_{0}+\Delta \alpha_{0,S}+\Delta \alpha \right)$$

where Δα is the attack angle induced by the post-critical vibration, which can be expressed as

(21) $$\Delta \alpha =\alpha \left(t\right)+\frac{\dot{h}\left(t\right)}{U}=a_{\alpha }\cos\varphi -\frac{a_{h}\omega_{t}}{U}\sin\left(\varphi +\Delta \beta \right)$$

where $\varphi =\omega_{t}t+\beta_{\alpha }$ is the torsional phase angle.

Substituting Equation (18b) into Equation (20) yields

(22) $$\begin{array}{l} k_{h}\Delta h_{0,L}=\frac{1}{2}\rho U^{2}BLC_{L}\left(\alpha_{0}+\Delta \alpha_{0,S}+\Delta \alpha \right)-\frac{1}{2}\rho U^{2}BLC_{L}\left(\alpha_{0}+\Delta \alpha_{0,S}\right)\\ \;\;\;\;\;\;\;\;=\frac{1}{2}\rho U^{2}BL\left({\frac{dC_{L}}{d\alpha_{0}}|}_{\alpha =\alpha_{0}^{*}}\Delta \alpha +{\frac{d^{2}C_{L}}{d\alpha^{2}}|}_{\alpha =\alpha_{0}^{*}}\frac{\Delta \alpha_{}^{2}}{2!}+{\frac{d^{3}C_{L}}{d\alpha^{3}}|}_{\alpha =\alpha_{0}^{*}}\frac{\Delta \alpha_{}^{3}}{3!}+\cdots \right) \end{array}$$

Take the average value of Equation (22) and combine Equation (21), we have

(23) $$\frac{1}{T}\int_{0}^{T}k_{h}\Delta h_{0,L}d\tau =\frac{\rho U^{2}BL}{2T}\int_{0}^{T}\left\{C_{L}\left[\alpha_{0}^{*}+a_{\alpha }\cos\varphi -\frac{a_{h}\omega_{t}}{U}\sin\left(\varphi +\Delta \beta \right)\right]-C_{L}\left(\alpha_{0}^{*}\right)\right\}d\tau$$

(24) $$\begin{array}{l} k_{h}\Delta h_{0,L}=\frac{1}{2}\rho U^{2}BL\left[\frac{C_{L}^{\left(2\right)}\left(\alpha_{0}^{*}\right)}{2!}\left(a_{\alpha }^{2}+\frac{a_{h}^{2}\omega_{t}^{2}}{U^{2}}\right)+\frac{C_{L}^{\left(4\right)}\left(\alpha_{0}^{*}\right)}{4!}\frac{3}{8}\left(a_{\alpha }^{4}+\frac{a_{h}^{4}\omega_{t}^{4}}{U^{4}}+\frac{2a_{\alpha }^{2}a_{h}^{2}\omega_{t}^{2}}{U^{2}}\right)+\cdots \right]\\ \;\;\;\;\;\;\;\;\;\;\;\approx \frac{1}{2}\rho U^{2}BL\left[\frac{C_{L}^{\left(2\right)}\left(\alpha_{0}^{*}\right)}{2!}a_{\alpha }^{2}+\frac{C_{L}^{\left(4\right)}\left(\alpha_{0}^{*}\right)}{4!}\frac{3}{8}a_{\alpha }^{4}+\frac{C_{L}^{\left(6\right)}\left(\alpha_{0}^{*}\right)}{6!}\frac{5}{16}a_{\alpha }^{6}\right] \end{array}$$

where $T=2\pi /\omega_{t}$ is the torsional vibration period. $C_{L}^{\left(n\right)}$ represents the nth-order derivative of aerostatic lift coefficient $C_{L}\left(\alpha \right)$ with respect to attack angle α. Note that in the approximation of Equation (24), the contribution of heaving velocity on the instantaneous attack angle is neglected, because the relative amplitude ratio of the two terms in Equation (21) during post-critical LCOs is

(25) $$\frac{a_{h}\omega_{t}}{a_{\alpha }U}=\frac{\pi a_{h}}{ba_{\alpha }U^{*}}=\frac{\pi \gamma }{U^{*}}$$

Considering the value of heave-torsion coupling ratio γ in Figure 9, we can find that the relative amplitude-ratio in Equation (24) lies in the range of 0.0393–0.157. Therefore, neglecting the second term of Equation (21) leads to an error less than 2.5% in the quadratic term in Equation (24) and even smaller errors in higher terms.

**Figure 16:**
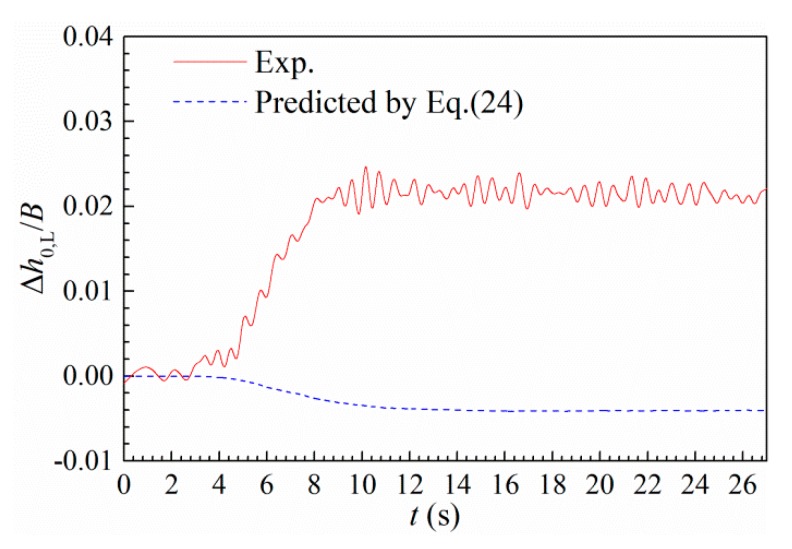
Time history of the coupled heaving drift during a post-critical LCO and the prediction by quasi-steady theory as Equation (24) (Case A1, U* = 5.365).

Figure 16 shows the calculated heaving drift $\Delta h_{0,L}$ during a large-amplitude post-critical LCO. One can find from the comparison of the calculated and test results that the quasi-steady theory fails to predict the experimental result. An unsteady theory needs to be built in the future study.

**Figure 17:**
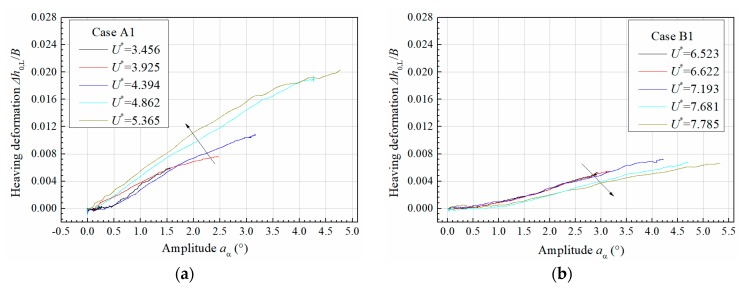
Evolution of additional heaving drift along with torsional amplitude for (**a**) attack angle 3° and (**b**) attack angle 0°.

Figure 17 presents the evolution of additional heaving drift $\Delta h_{0,L}$ with torsional amplitude. It can be found that the additional heaving drift $\Delta h_{0,L}$ all increases with torsional amplitude in an approximately linear manner. The evolution pattern with reduced velocity is very sensitive to initial attack angle. For positive attack angle 3°, higher reduced velocity corresponds to a more significant coupling of additional heaving drift, whereas for attack angle 0°, the coupling of heaving drift becomes slighter in higher reduced velocity. Note that the measured $\Delta h_{0,L}$ also includes high-frequency fluctuation as indicated in Figure 16; however, from an engineering point of view, the slow-varying component is the major concern and thus plotted in Figure 17 by smoothing out the high-frequency fluctuation.

## 3. Measurement of Nonlinear Aerodynamic Force

### 3.1. A Novel Measurement Technique

**Figure 18:**
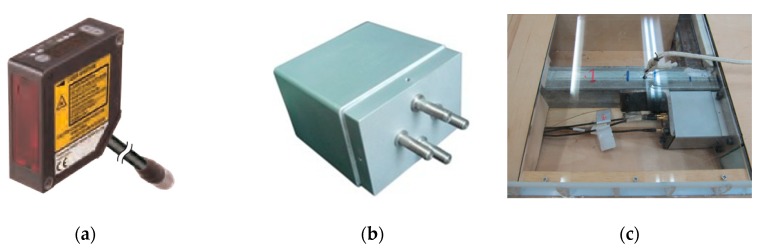
The dynamic sensors used in the measurement. (**a**) Laser displacement sensor; (**b**) force balance; (**c**) installation inside the sectional model.

A novel wind-tunnel technique was adopted in this study to measure the aerodynamic lift and torsional moment during post-critical LCOs with high precision [7,12]. As sketched in Figure 2, the outer ‘coat’ of the middle segment was isolated from the other parts and connected to internal rigid frame of the sectional model through 4 force balances (Figure 18). Hence, only the dynamic force acting on the middle ‘coat’ was measured. The ‘coat’ was made of thin wooden plates and stiffened by thin-walled duralumin to reduce its mass as possible. Therefore, the inertia force component was significantly reduced. The mass $m_{s}$ and moment of inertia $J_{s}$ of the middle ‘coat’ segment were 1.824 kg/m and 0.038 kg·m^2^/m, respectively, which only account for about 32% and 28% of the total effect values of the spring-suspended system.

The force balances were elaborately manufactured with high sensitivity. It is a kind of piezoelectric-type three-component force balance (Figure 18b); it is small in size, i.e., 0.035 × 0.05 × 0.05 m to be installed inside sectional model. The mass of each balance is about 0.128 kg. The linear measuring range is 12 N (shear force) and 0.9 N·m (torque) with a basic error <4.57% F.S. The 4 force balances were installed inside the sectional model (Figure 18c), and together with the 4 laser displacement sensors (Figure 18a) were connected to a 24 bits resolution acquisition system, which enables a synchronous measurement of aerodynamic force and displacement.

**Figure 19:**
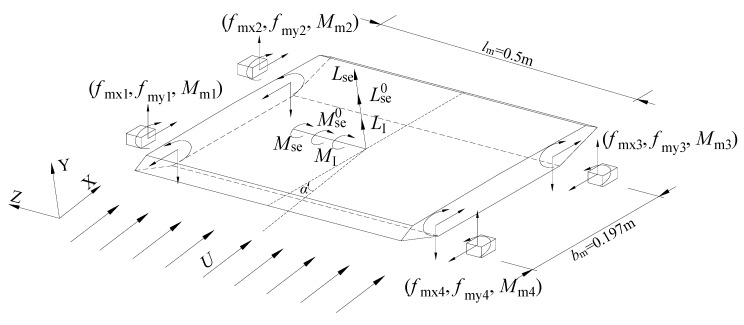
Definition and notation of the measurement technique of aerodynamic force.

The self-excited lift and torsional moment can then be extracted from the measured force signals. Figure 19 shows the dynamic equilibrium condition of middle ‘coat’ segment. The oscillating model immersed in flowing air is acted by four types of dynamic force, i.e., self-excited force, inertial force, non-wind-induced force and the dynamic actions by the 4 balances as plotted in Figure 19. Note that the static forces, which include self-weight, aerostatic force and static actions by force balances, are balanced with each other around the zero-vibrating position. Therefore, the static forces are not shown in Figure 19, and the following equations can be established around the zero position, which are

(26a) $$M_{\mathrm{se}}(t)=M_{\mathrm{ms}}(t)-M_{\mathrm{se}}^{0}(t)-M_{I}(t)$$

(26b) $$L_{\mathrm{se}}(t)=L_{\mathrm{ms}}(t)-L_{\mathrm{se}}^{0}(t)-L_{I}(t)$$

where $M_{I}(t)=-J_{s}\cdot \ddot{\alpha }\left(t\right)$ is the inertial moment per unit length being $\ddot{\alpha }$ the angular acceleration. $L_{I}(t)=-m_{s}\cdot \ddot{h}\left(t\right)$ is the heaving inertial lift per unit length being $\ddot{h}$ the heaving acceleration. $M_{\mathrm{ms}}$ and $L_{\mathrm{ms}}$ are respectively the resultant torsional moment and lift by the force sensors

(27a) $$M_{\mathrm{ms}}(t)=\left[\left(M_{m1}+M_{m2}-M_{m3}-M_{m4}\right)+\left(F_{\mathrm{my}1}-F_{\mathrm{my}2}+F_{\mathrm{my}3}-F_{\mathrm{my}4}\right)\times b_{m}/2\right]/l_{m}$$

(27b) $$L_{\mathrm{ms}}(t)=\left(F_{\mathrm{my}1}+F_{\mathrm{my}2}+F_{\mathrm{my}3}+F_{\mathrm{my}4}\right)\times \cos\alpha_{0}^{*}+\left(-F_{\mathrm{mx}1}-F_{\mathrm{mx}2}+F_{\mathrm{mx}3}+F_{\mathrm{mx}4}\right)\times \sin\alpha_{0}^{*}$$

where $M_{mi}$,$F_{\mathrm{my}i}$,$F_{\mathrm{mx}i}$(*i* = 1,2,3,4) represent the measured force signals by each force balance as illustrated in Figure 19. $l_{m}$ = 0.5 m is the length of middle ‘coat’ segment. $b_{m}$ = 0.197 m is the transverse distance of force sensors. 

$M_{\mathrm{se}}^{0}$ and $L_{\mathrm{se}}^{0}$ are non-wind-induced force due to the mutual action of vibrating model and its surrounding air. Non-wind-induced force has the effect of added damping and added mass on the vibration system, which is also called as “virtual mass effect” by Wilkinson [19]. The non-wind-induced forces have been extensively discussed in previous studies [7,20]. $M_{\mathrm{se}}^{0}$ and $L_{\mathrm{se}}^{0}$ are expressed as

(28a) $$M_{\mathrm{se}}^{0}(t)=-J_{0}\cdot \ddot{\alpha }\left(t\right)-c_{\alpha 0}\cdot \dot{\alpha }\left(t\right)$$

(28b) $$L_{\mathrm{se}}^{0}(t)=-m_{0}\cdot \ddot{h}\left(t\right)-c_{h0}\cdot \dot{h}\left(t\right)$$

where $J_{0}$ and $c_{\alpha 0}$ are respectively added mass moment of inertia and added torsional damping coefficient. $m_{0}$ and $c_{h0}$ are respectively added mass and added heaving damping coefficient. The identification of $J_{0}$, $m_{0}$, $c_{\alpha 0}$ and $c_{h0}$ are performed from the free decay responses in still air and one can refer to Gao and Zhu [7,20] for more details.

### 3.2. Aerodynamic Nonlinearity

**Figure 20:**
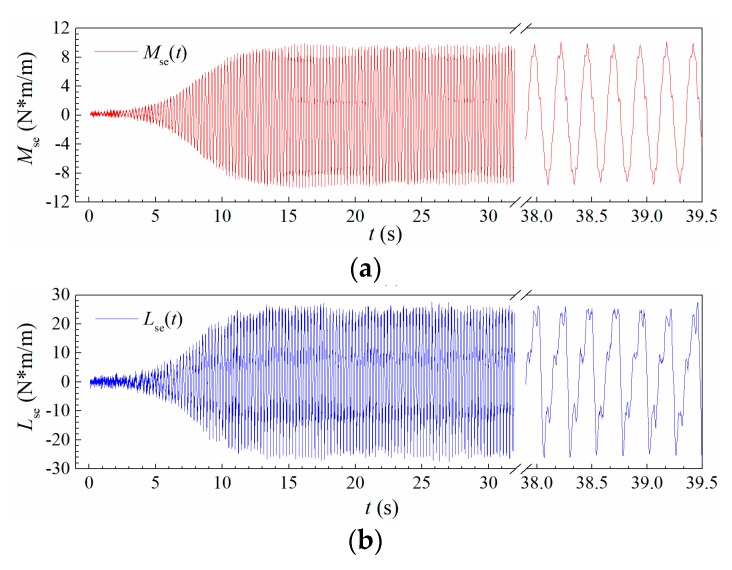
Time histories of the measured aerodynamic (**a**) torsional moment and (**b**) lift (Case B1, U* = 7.785).

Figure 20 shows the time histories of the measured self-excited torsional moment $M_{\mathrm{se}}$ and lift $L_{\mathrm{se}}$ during a post-critical LCO for Case B1 when U* = 7.785. The vibration curves are also self-limiting similar to displacement responses in Figure 6c, whereas, their curve shapes during the steady-amplitude stage are obviously distorted from a pure sinusoidal wave, especially for self-excited lift $L_{\mathrm{se}}$, indicating that there exists significant aerodynamic nonlinearity.

**Figure 21:**
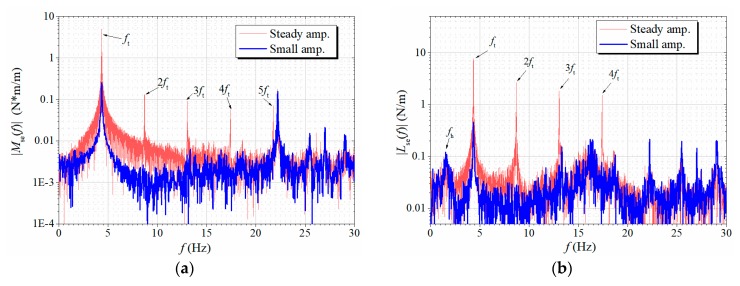
Amplitude spectra of the measured aerodynamic (**a**) torsional moment and (**b**) aerodynamic lift during a post-critical LCO (Case B1, U* = 6.623).

Figure 21 further displays the amplitude spectra of the measured $M_{\mathrm{se}}$ and $L_{\mathrm{se}}$. One can find the existence of significant higher-order harmonic components in both spectra in the steady-amplitude stage, whereas during small amplitude stage before the post-critical LCO build up, the spectra only contain the fundamental frequency, which means that the aerodynamic nonlinearity increases with vibration amplitude. Besides, in the lift spectrum, one can observe a slight component of heaving frequency, which does not increase during the post-critical LCO process, which again confirms that the observed post-critical LCOs only occur in the torsional mode and the heaving mode is always stable.

To check the accuracy of the measured self-excited force $M_{\mathrm{se}}$ and $L_{\mathrm{se}}$, the time histories of the measured force signals were directly applied on the vibration system of sectional model to predict the post-flutter responses. The calculated responses were then compared with experimental ones. The calculation of post-flutter responses was based on the governing dynamic equations around zero static positions, which can be expressed as

(29a) $$\ddot{\alpha }+2\xi_{\alpha 0}\omega_{t0}\dot{\alpha }+\omega_{t0}^{2}\alpha =M_{\mathrm{se}}\left(t\right)/\left(I+J_{0}\right)$$

(29b) $$\ddot{h}+2\xi_{h0}\omega_{h0}\dot{h}+\omega_{h0}^{2}h=L_{\mathrm{se}}(t)/\left(m+m_{0}\right)$$

The solving of Equation (29) was performed by an explicit Newmark-β method through an iteration procedure. During the calculation, the amplitude-dependent effect of structural damping ratios ($\xi_{\alpha 0}$,$\xi_{h0}$) and frequencies ($\omega_{t0}$,$\omega_{h0}$) were also considered, for which one can refer to the previous studies by Gao and Zhu [6].

**Figure 22:**
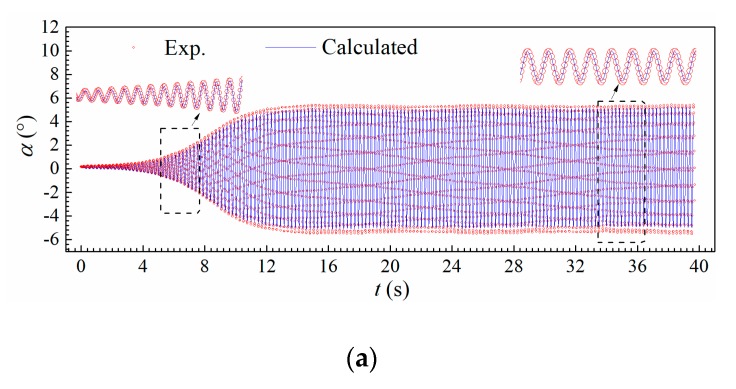

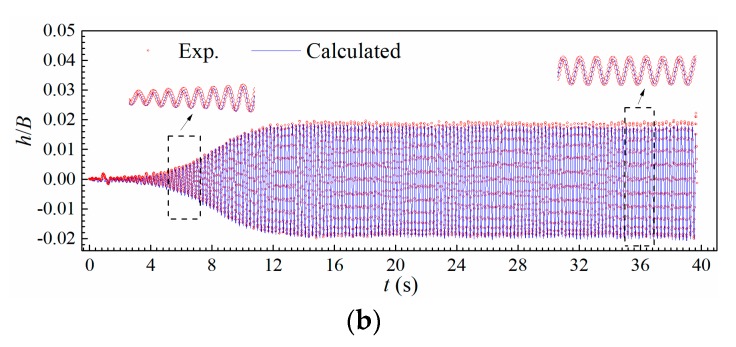
Comparison of tested and predicted (**a**) torsional and (**b**) heaving displacement during a post-critical LCO by the measured force signals (Case B1, U* = 7.785).

Figure 22 shows the calculated post-critical LCOs for Case B1 when U* = 7.785. It is found that the calculated results are in good agreement with experimental ones, where the discrepancies are about −4.8% and −6.8%, respectively. Thus, the measured self-excited force signals are validated to be reliable and can further be used for analyzing the energy mechanism during post-critical LCOs. 

### 3.3. Energy Evolving Mechanism

**Figure 23:**
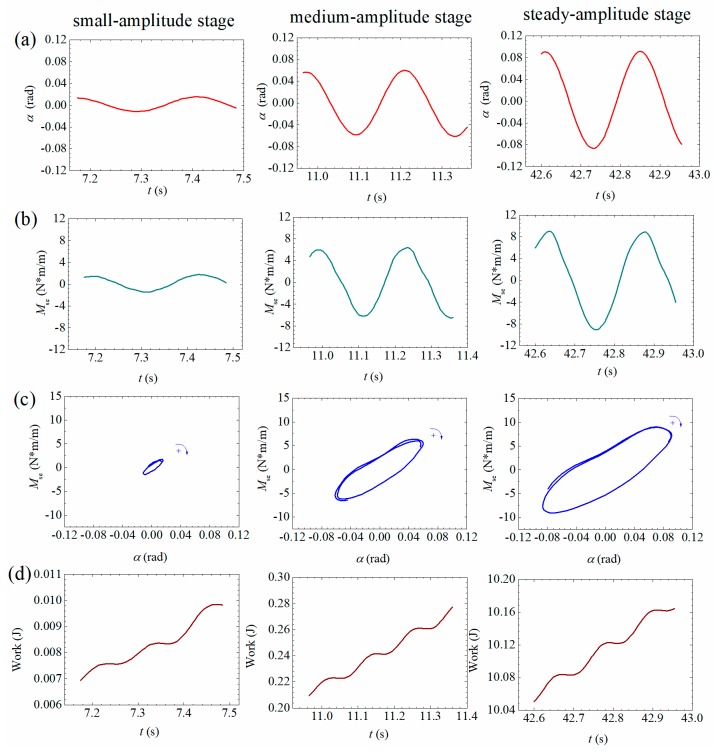
Evolutionary energy mechanism during a post-critical LCO in the torsional DOF (Case B1, U* = 7.785). (**a**) Torsional dispalcement; (**b**) measure of aerodynamic torsional moment; (**c**) hysteresis loops; (**d**) accumulative works.

**Figure 24:**
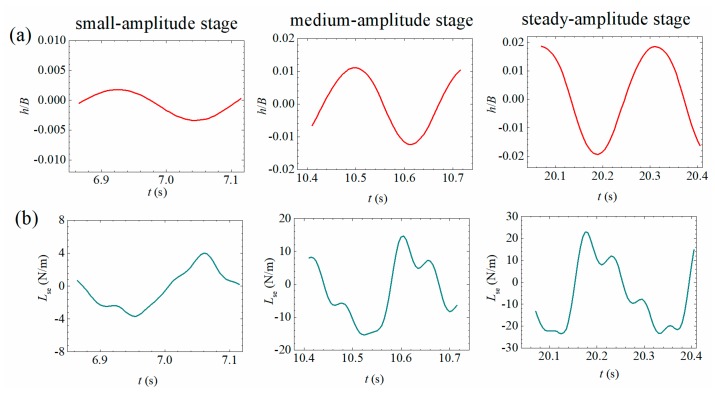

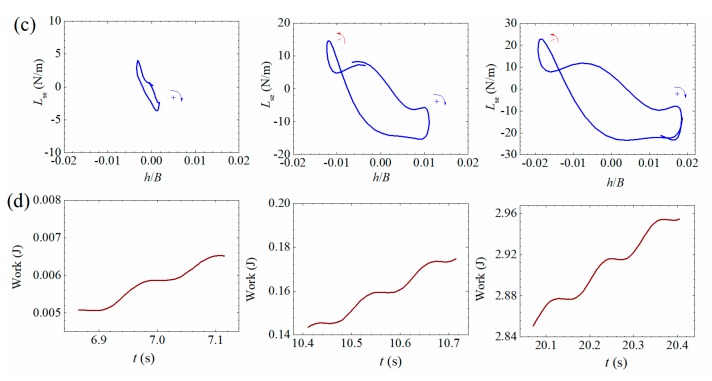
Evolutionary energy mechanism during a post-critical LCO in the heaving DOF (Case B1, U* = 7.785). (**a**) Heaving displacement; (**b**) measure aerodynamic lift; (**c**) hysteresis loops; (**d**) accumulative works.

Based on the measured signals of self-excited force, the energy evolving mechanism during post-critical LCOs can be analyzed by using the evolution of hysteresis loops. Three typical vibration curves during a LCO build-up process of Case B1 (Figure 6c) are chosen in Figure 23a and Figure 24a. As shown in Figure 23 and Figure 24, the left column corresponds to the small amplitude stage; the middle column corresponds to the medium amplitude stage and the right column is related with the steady-amplitude stage.

As discussed by Diana et al. [21] and Zhang et al. [14], a hysteresis loop is defined as the curve of aerodynamic force versus the corresponding displacement. The area enclosed by a hysteresis loop equals the accumulative work done by the aerodynamic force in each vibrating period. A clockwise loop indicates positive aerodynamic work and thus the vibrating model absorbing energy from the flowing air, and an anticlockwise loop indicates negative work and thus the vibrating model dissipating energy. The accumulative aerodynamic work is defined as the integration of the transient aerodynamic power with respect to time, which is

(30a) $$W_{M\mathrm{se}}=\int_{t_{0}}^{t}M_{\mathrm{se}}(\tau )\dot{\alpha }d\tau$$

(30b) $$W_{L\mathrm{se}}=\int_{t_{0}}^{t}L_{\mathrm{se}}(\tau )\dot{h}d\tau$$

where $t_{0}$ represents the time point when post-critical LCO starts. $W_{M\mathrm{se}}$ is the accumulative work done by the measured self-excited moment on the torsional heaving DOF. $W_{L\mathrm{se}}$ is the accumulative work done by the measured self-excited lift on the heaving DOF.

Figure 23c and Figure 24c present the typical hysteresis loops during a LCO build-up process. One can find that the loops are both clockwise in small amplitude stages indicating absorbing energy. The enclosed area increases with the building up of vibration amplitude. The corresponding accumulated works are plotted in Figure 23d and Figure 24d. It can be found that the accumulative aerodynamic work in torsional/heaving DOF both increases with the enlarging area of clockwise loops. Note that the positive aerodynamic works in the steady amplitude stage compensate the amount of energy dissipated by the mechanical damping in torsional/heaving DOF.

It is also noticed that the shapes of hysteresis loops are increasingly distorted from a pure ellipse during the build-up process of a post-flutter LCO. Because a pure ellipse loop corresponds to a linear case, whereas with the increase of vibration amplitude, the aerodynamic nonlinearity becomes more significant as indicated in Section 3.2. As a result of the higher-order harmonic components, the hysteresis loop evolves into a distorted cycle for the torsional DOF and an 8-shape cycle for the heaving DOF. The 8-shape hysteresis loop indicates that the work by self-excited lift changes from negative to positive in a vibrating period. However, Zhang et al. [14] recently pointed out through a CFD calculation that the distortion by higher-order harmonic components does not influence the resultant accumulative aerodynamic work.

## 4. Discussion of the Results

The classical linear flutter theory is not applicable to predict post-flutter instabilities. Because the observed post-flutter responses are characterized by a nonlinear self-limiting LCO with time-varying damping ratio as indicated by Figure 13a, whereas the linear flutter theory is based on a small-amplitude assumption and only considers the constant aerodynamic damping and stiffness [1]. Thus, the predicted linear flutter instability is a ‘hard’ flutter with the vibration amplitude increasing exponentially with time. The aerodynamic nonlinearity under large amplitude has a stabilizing effect, and the self-limiting behavior is beneficial to the safety of long-span bridges. 

Figure 13a, Figure 23 and Figure 24 clearly demonstrate that the aerodynamic nonlinearities instead of mechanical nonlinearities are responsible for the observed nonlinear post-flutter instabilities. Because according to the previous studies [15] on the mechanical nonlinearities of spring-suspended sectional model systems, the mechanical damping ratio increases with amplitude resulting in a more significant energy-dissipating effect. The mechanical stiffness generally exhibits a slight behavior of soft spring, which only slightly changes the instantaneous phase of vibration response.

The coupling of heaving static deformation and post-critical LCO discussed in Section 2.6 suggests that large-amplitude torsional vibration may possibly induce static divergence for long-span bridges, because the heaving static deformation plays an important role in the occurrence of aerostatic divergence for cable-supported bridges, especially suspension bridges [22]; the additional upward deformation during large-amplitude vibration will change the tension state of main cables and will inevitably reduce the structural torsional stiffness provided by the cable system. It is also indicated from Figure 16 that classical quasi-steady theory is not applicable. Little attention has been paid to this phenomenon; however, it deserves more investigation in the future study.

A nonlinear theory is necessary to predict the nonlinear post-flutter responses. It is the future work of this study to establish a new coupled self-excited force model to fully consider the aerodynamic nonlinearities based on the measured force signals. The nonlinear self-excited force model should be able to simulate the amplitude-dependent behaviors of torsional mode (heave-torsion coupling effect), the aerodynamic damping and the coupling of heaving aerostatic deformation.

## 5. Conclusions

The nonlinear behaviors of flutter instabilities on a typical box-type bridge deck were investigated through a series of wind-tunnel sectional model tests. The dynamic displacement responses together with nonlinear aerodynamic forces were synchronously measured by using laser displacement sensors and piezoelectric force balances. Major concluding remarks are drawn as follows:

(1) Beyond linear flutter boundary, the investigated flat closed-box bridge deck (side ratio 9.14) lost its torsional stability around zero position and exhibited nonlinear post-critical LCOs in post-flutter states under attack angle 3° and 0°. The post-critical LCOs were heave-torsion coupled vibration in the torsional mode, which is a ‘soft’ type flutter with a gradual increase of stable amplitudes with reduced velocities. The degree of heave-torsion coupling increased with reduced velocity.

(2) The heave-torsion coupled LCO vibrated in a complex torsional mode. The mode modulus and phase angle both evolved with amplitude. The damping ratios exhibited a significant amplitude-dependent effect. Whilst the vibration frequency contained fast-fluctuating components, and its long-term trend slightly evolved with amplitude.

(3) An obvious coupling of post-critical LCOs and heaving static deformation was observed. The coupling effect was significant under attack angle 3° but relatively slight for attack angle 0°. The evolution of coupling behavior with reduced velocity was sensitive to attack angle. Classical quasi-steady theory, which is applicable for predicting the static deformation under small amplitude, was found to be unsuitable, and a new theory is needed.

(4) The measured signals of aerodynamic torsional moment and lift were found to contain significant higher-order harmonic components. The post-critical LCOs were well predicted by the measured force signals. The energy mechanisms were closely related with the evolving shapes of hysteresis loops of the measured aerodynamic force versus dynamic displacement.

## Figures and Tables

**Table 1:** Main dynamic parameters of aeroelastic tests. *α*_0_ is wind attack angle in still air; *f*_t0_ and *f*_h0_ are linear torsional and heaving frequency in still air, respectively. *ξ*_α0_ and *ξ*_h0_ are linear mechanical damping ratios of the torsional and heaving modes. *J*_m_ and *m* are the effective moment of inertia and mass per unit length, respectively, in the elastically supported vibration system.

#.	*α*_0_	*ξ*_α0_	*ξ*_h0_	*f*_t0_	*f*_h0_	*J*_m_	*m*
	(°)	(%)	(%)	(Hz)	(Hz)	(kg·m^2^/m)	(kg/m)
A1	3	0.117	0.325	4.834	1.773	0.136	5.774
A2	3	0.178	0.490	4.827	1.789	0.139	5.774
B1	0	0.101	0.343	4.836	1.773	0.136	5.774
B2	0	0.209	0.521	4.822	1.788	0.138	5.774
C1	−3	0.0930	0.566	4.820	1.774	0.136	5.774
C2	−3	0.197	0.818	4.825	1.789	0.139	5.774

## Notation

$a_{0}$	a constant related with initial condition	Re	Reynolds number
$a_{\alpha },\;a_{h}$	torsional, heaving amplitude	t	time
$A_{i}^{*}$,$H_{i}^{*}$	flutter derivatives	$t_{0}$	starting time point
b	half width of a cross section	T	torsional period,$2\pi /\omega_{t}$
$b_{m}$	distance of force sensors	U	wind velocity
B, D	width/depth of a cross section	$U_{\mathrm{cr}}^{*}$	non-dimensional linear flutter boundary
$c_{\alpha 0}$, $c_{h0}$	added damping coefficients	$U_{}^{*}$	reduced wind velocity, U/fB
$C_{D},\;C_{L},\;C_{M}$	drag, lift and moment coefficients	$U_{h2}$,$V_{h2}$	heaving mode parameters
$C_{L}^{\left(n\right)}$	nth-order derivative of lift coefficient	$U_{\alpha 2}$,$V_{\alpha 2}$	torsional mode parameters
$D_{0}$	effective windward height	$U_{h2}^{\#}$,$V_{h2}^{\#}$	normalized mode parameter
e	distance of spring fixed points	$W_{M\mathrm{se}}$	work by self-excited moment
$f_{t0},\;f_{h0}$	frequencies in still air	$W_{L\mathrm{se}}$	work by self-excited lift
$f_{t},\;f_{h}$	torsional and heaving frequency	$\alpha ,\;\dot{\alpha },\ddot{\alpha }$	torsional angle, angular velocity and acceleration
$F_{D}$,$F_{L}$,M	aerodynamic drag force, lift force and torsional moment per unit length	$\alpha_{0}$	initial wind angle of attack
$h,\;\dot{h},\;\ddot{h}$	heaving displacement, velocity and acceleration	$\alpha_{0}^{*}$	static attack angle under flowing air conditions
$h_{\mathrm{se}}$,$h_{0}$	pure heaving displacement, heaving static deformation	$\beta_{\alpha }$,$\beta_{h}$	torsional and heaving phase angle
$h_{\max}$,$h_{\min}$	upper, lower heaving envelopes	γ	heave-torsion coupling ratio
$h_{\mathrm{rms}}$,$\alpha_{\mathrm{rms}}$	heaving, torsional root-mean-squares	$\xi_{\alpha 0},\;\xi_{h0}$	mechanical damping ratios in still air
$H_{\mathrm{wt}}$	height of wind-tunnel test section	$\xi_{t}$	torsional damping ratio
$I_{u}$	turbulence intensity	$\xi_{t}$	total damping ratio of the torsional mode
$J_{m}$	effective mass moment of inertia	$\xi_{s}$	amplitude-dependent structural damping ratio
$k_{h}$,$k_{\alpha }$	elastic heaving, torsional stiffness	$\xi_{\mathrm{se}}$	aerodynamic damping ratio induced by self-excited force
K	reduced frequency	ρ	air density
$l_{m}$	length of middle ‘coat’ segment	φ	torsional phase angle
L	axial length of a sectional model	$\omega_{t}$	circular frequency of torsional mode
$L_{I}$	inertial lift per unit length, $-m_{s}\cdot \ddot{h}$	$\omega_{t0}$,$\omega_{h0}$	torsional, heaving circular frequencies in still air
$L_{\mathrm{se}},\;M_{\mathrm{se}}$	self-excited lift and torsional moment	$\Delta h_{0}$	total drift of the heaving zero position
m	effective mass per unit length	$\Delta h_{0,S}$	heaving deformation under small amplitude
$m_{0}$,$J_{0}$	added mass, mass moment of inertia	$\Delta h_{0,L}$	heaving deformation under large amplitude
$m_{s}$,$J_{s}$	mass, moment of inertia of middle ‘coat’	Δα	effective attack angle induced vibration
$M_{I}$	inertial moment per unit length	$\Delta \alpha_{0,S}$	drift of static attack angle relative
$M_{\mathrm{ms}}$,$L_{\mathrm{ms}}$	resultant torsional moment and lift	Δβ	phase difference between heaving and torsional displacement
$M_{mi}$, $F_{\mathrm{my}i}$$F_{\mathrm{mx}i}$	measured force signals	$\varphi_{t}$	vector of torsional mode
$M_{\mathrm{se}}^{0}$,$L_{\mathrm{se}}^{0}$	non-wind-induced force per unit length	Real()	the real part of a complex value
